# Liensinine Inhibits Osteosarcoma Growth by ROS-Mediated Suppression of the JAK2/STAT3 Signaling Pathway

**DOI:** 10.1155/2022/8245614

**Published:** 2022-01-25

**Authors:** Fei Jia, Yu Liu, Xinyu Dou, Chuanchao Du, Tianli Mao, Xiaoguang Liu

**Affiliations:** ^1^Department of Orthopedics, Peking University Third Hospital, North Garden Street No. 49, Haidian District, Beijing 100191, China; ^2^Beijing Key Laboratory of Spinal Diseases, Haidian District, Beijing 100191, China; ^3^Engineering Research Center of Bone and Joint Precision Medicine, Haidian District, Beijing 100191, China

## Abstract

Osteosarcoma (OS) is the most common malignancy of bone. Liensinine exerts antitumor effects on cancers of the colon, breast, and gallbladder. However, its antitumor activity in OS remains unclear. This study is aimed at investigating the efficacy of liensinine against OS and the underlying mechanism of action. Cell proliferation, apoptosis, and cycle arrest in OS were detected using the Cell Counting Kit-8 (CCK-8), colony formation, and flow cytometry assays, respectively. The production of reactive oxygen species (ROS), glutathione (GSH) and glutathione disulfide (GSSG) concentrations, and mitochondrial membrane potential (MMP) of OS cells were measured by flow cytometry, colorimetry, and JC-1 staining. The expressions of factors related to apoptosis, cell cycle, and activation of the JAK2/STAT3 pathway were determined by Western blotting. To examine the potential role of ROS, an antioxidant (N-acetyl cysteine, NAC) was used in combination with liensinine. *In vivo*, we generated a xenograft mouse model to assess its antitumor efficacy. Tissue level expressions of factors related to apoptosis and activation of the JAK2/STAT3 pathway were assessed by immunohistochemistry or Western blotting. Liensinine inhibited the proliferation and induced G0/G1 phase arrest and apoptosis of OS cells in a dose-dependent manner. Additionally, liensinine promoted intracellular ROS production, enhanced the GSSG/GSH ratio, and induced MMP loss and ROS-mediated suppression of the JAK2/STAT3 pathway. NAC significantly attenuated the liensinine-induced antitumor activities and activated the JAK2/STAT3 pathway. *In vivo*, liensinine effectively inhibited the OS growth and promoted apoptosis; however, it had no negative effect on the internal organs. In conclusion, liensinine-induced ROS production could suppress the activation of the JAK2/STAT3 pathway and inhibit the OS growth both *in vivo* and *in vitro*. Our findings provided a new rationale for subsequent academic and clinical research on OS treatment.

## 1. Introduction

Osteosarcoma (OS) is a malignant tumor originating from mesenchymal tissues, accounts for 20% of the primary malignant bone acanthomas; it is the most common primary malignant bone tumor in adolescents [1]. OS is characterized by high malignancy, rapid growth, early metastasis, and poor prognosis. It is the leading cause of cancer-related death among children and adolescents [2, 3]. At present, surgery combined with multiagent chemotherapy is the standard treatment regime for OS [2, 4]. However, long-term chemotherapy causes many irreversible systemic side effects, including cardiotoxicity, secondary malignancies, neurotoxicity, and infertility. In addition, physical disability caused by surgical procedures has a great impact on the mental health and quality of life of the patients [2, 5]. Therefore, the development of novel anti-OS drugs is important for effectively inhibiting tumor progression and prolonging the survival duration of patients.

Liensinine, a natural isoquinoline alkaloid, is isolated from the seed embryo of Nelumbo nucifera Gaertn [6]. Multiple biological effects of liensinine are reported in recent pharmacological studies, including antihypertension and antiarrhythmias, as well as anticancer properties [6–8]. For example, liensinine can induce apoptosis and growth cessation of gallbladder cancer [9]. It can also induce apoptosis of colorectal cancer by stimulating mitochondrial dysfunction and sensitize breast cancer cells towards chemotherapy through DNM1L-mediated mitochondrial fission [6, 10]. However, unlike the aforementioned cancer types, OS is distinct in both origin and biological behavior. The role of liensinine in OS remains unknown and warrants investigation.

Reactive oxygen species (ROS) are the by-products of oxidative stress, which include peroxides, superoxides, hydroxyl radicals, and singlet oxygen [11]. Accumulating evidence demonstrates that several anticancer drugs, such as Glaucocalyxin A, metformin, cisplatin, isoalantolactone, and docetaxel, can induce apoptosis and proliferation cessation of cancer cells by intracellular ROS generation [11–13]. According to previous studies, the inhibitory effect of liensinine on gastric cancer cells is achieved by inducing ROS production [14]. In addition, other similar dibenzyl tetrahydroisoquinoline alkaloids, such as cepharanthine or tetrandrine, can also induce ROS-mediated apoptosis of tumor cells [15, 16]. Typically, tumor cells can withstand oxidative stress by maintaining the balance between oxidation and antioxidation levels; however, antineoplastic drugs can fatally break the redox balance not just by increasing oxidant accumulation but also by interfering with the antioxidant production [11]. The role of liensinine in the oxidation/antioxidation system of OS cells is unknown.

In this study, we aimed to investigate the antitumor effect of liensinine on OS and further explore its underlying mechanism of action, so as to elucidate the regulation of ROS on the JAK2/STAT3 pathway.

## 2. Materials and Methods

### 2.1. Cell Culture

The source and culture conditions of human normal osteoblasts, hFOB 1.19, and human OS cell lines SaOS-2, MG-63, 143B, and U-2OS were the same as described in our previous study [17].

### 2.2. Drug Treatment

Liensinine (HY-N0484) with 99.89% purity was obtained from MedChemExpress (Princeton, New Jersey, USA). N-Acetyl cysteine (NAC, ST1546), an antioxidant and a ROS inhibitor, was purchased from Beyotime (Shanghai, China). For liensinine treatment, the cells were cultured in the medium containing 0 *μ*M, 5 *μ*M, 10 *μ*M, 20 *μ*M, 40 *μ*M, or 80 *μ*M liensinine for 24 h; following which, they were observed under an inverted microscope (Leica, Wetzlar, Germany). For cotreatment with liensinine and NAC, the cells were treated with 80 *μ*M liensinine for 24 h following a 2 h pretreatment with 5 mM NAC.

### 2.3. Cell Viability Assay

The Cell Counting Kit-8 (CCK-8) (C0038, Beyotime) was used to detect the inhibitory effect at different concentrations of liensinine (0 *μ*M, 5 *μ*M, 10 *μ*M, 20 *μ*M, 40 *μ*M, or 80 *μ*M) as described previously [18].

### 2.4. Colony Formation Assay

1000 cells per well were plated onto 6-well plates (each well contained 2 mL medium supplemented with 10% FBS). After adherence for 24 h, the cells were treated with 0 *μ*M, 40 *μ*M, and 80 *μ*M liensinine for 24 h; following which, the medium was replaced with the complete medium. The cells were cultured for 14 days; subsequently, they were fixed with 4% ice-cold paraformaldehyde, stained with 0.1% crystal violet, and washed thrice with PBS (P1010, Solarbio, Beijing, China). Images of cell colonies were captured by a camera (Alpha 7R IV, SONY, Tokyo, Japan), and the cell colony numbers were analyzed using the Image J software.

### 2.5. Flow Cytometric Analysis of Apoptosis

The apoptosis was evaluated by flow cytometry (FCM) using an Annexin V-FITC/PI Apoptosis Detection Kit (C1062L, Beyotime). After SaOS-2 and 143B cells were treated with liensinine or cotreated with NAC and liensinine in 6-well plates for 24 h, approx. 1 × 10^6^ cells/well were harvested and detected as described previously [19].

### 2.6. Flow Cytometric Analysis of Cell Cycle Distribution

The cell cycle distribution of cells was evaluated by FCM using a Cell Cycle Detection Kit (C1052, Beyotime). After SaOS-2 and 143B cells were treated with liensinine for 24 h, approx. 1 × 10^6^ cells/well were harvested and washed twice with PBS. The resuspended cells were then fixed with prechilled 70% ethanol (E111994, Aladdin, Shanghai, China) for 2 h at 4°C. Following this, the collected cells were incubated with 500 *μ*L PI/RNase A staining fluid for 30 min at 37°C in dark. The cell cycle distribution was detected and analyzed on a CytoFLEX flow cytometer (Beckman Coulter, Brea, CA, USA) with 488 nm excited wavelength.

### 2.7. Flow Cytometric Analysis of ROS

The ROS production in the cells was evaluated by FCM using a DCFH-DA probe-based ROS Detection Kit (S0033, Beyotime). DCFH-DA was diluted with FBS-free medium to a final concentration of 10 *μ*M. After SaOS-2, 143B, and hFOB 1.19 cells were treated with liensinine or cotreated with NAC and liensinine for 24 h, the cells (1 × 10^6^) were harvested and washed twice with PBS. The cells were then incubated with 500 *μ*L of 10 *μ*M DCFH-DA for 20 min in a 37°C incubator and washed thrice with FBS-free medium. Finally, the ROS in the cells was detected and analyzed on a CytoFLEX flow cytometer with 488 nm excited wavelength.

### 2.8. Colorimetric Analysis of Glutathione (GSH) and Glutathione Disulfide (GSSG)

The level of GSH and GSSG in the cells was evaluated by colorimetry using a GSH and GSSG Detection Kit (S0053, Beyotime). After SaOS-2 and 143B cells were treated with liensinine or NAC, the cells were collected and washed with PBS. 30 *μ*L of protein removal reagent M solution was added. The samples were then subjected to two rapid freeze-thaws using liquid nitrogen and water at 37°C. After the samples were placed at 4°C for 5 min, they were centrifuged for 10 min at 10,000 × g, and the supernatant was collected for the determination of total glutathione. In addition, part of the above supernatant was pipetted and added with the GSH clearance auxiliary solution as well as the GSH clearance working solution at the ratios of 100 *μ*L : 20 *μ*L and 100 *μ*L : 4 *μ*L. The solutions were mixed by vortex and reacted for 60 min at room temperature to obtain samples for GSSG determination. In a 96-well plate, 10 *μ*L of the supernatant was mixed with 150 *μ*L of the prepared total glutathione detection working solution and incubated at room temperature for 5 min, followed by the addition of 50 *μ*L of 0.5 mg/mL NADPH. After 25 minutes of reaction, the absorbance of each well was measured by a microplate reader at 412 nm wavelength. GSSG was determined in the same way. The GSH and GSSG levels were further calculated according to the kit manufacturer's protocol and the formula: GSH = total glutathione − GSSG × 2.

### 2.9. JC-1 Staining

The mitochondrial membrane potential (MMP) of the cells was evaluated by a JC-1 Kit (C2006, Beyotime). After SaOS-2 and 143B cells were treated with liensinine or NAC in the 6-well plates for 24 h, the medium was discarded and 1 mL JC-1 working solution was added to the wells, and cells were incubated for 20 min at 37°C. The cells were then washed twice with 1× JC-1 staining buffer. After adding 1 mL PBS per well, the fluorescence signal of the cells was observed under an inverted fluorescence microscope (DMI8, Leica) at ×200 magnification. Polymers were excited by green light, monomers were excited by blue light, and merged images were collected.

### 2.10. *In Vivo* Assay

The animal study design was approved by the ethics committee of Peking University Third Hospital (No. IRB00006761-2016048) and complied with the ARRIVE guidelines. All experiments with animals were performed in the Department of Laboratory Animal Science, Peking University Health Science Center. Eighteen 4-week-old BALB/c nude female mice weighing 18 ± 2 g were purchased from Beijing WeiTongLiHua Laboratory Animal Technology Co., Ltd. The mice were kept in a specific pathogen free (SPF) environment and provided an ad libitum diet. After feeding for five days, the mice adapted to the environment; next, 143B cells at a density of 2 × 10^6^/100 *μ*L were subcutaneously injected into the right axilla of each mouse. When the tumor volume reached 100 mm^3^, the mice were randomly divided into three groups: (1) sham treatment (*n* = 6), (2) liensinine-L treatment (*n* = 6), and (3) liensinine-H treatment (*n* = 6). The mice in the three groups were orally administered with 200 *μ*L PBS, 15 mg/kg liensinine solution, or 30 mg/kg liensinine solution, respectively, once every two days for 14 days. The tumor sizes and body weights were measured every two days with the volume calculation formula: *L* × *W*2/2 (*L*, the long diameter of the tumor and *W*, the short diameter of the tumor). On the last day, the mice were sacrificed. Subcutaneous tumors from each mouse were excised and weighed; one portion was immediately frozen for Western blot analysis, while another was preserved in 4% paraformaldehyde for histopathological analysis. In addition, the heart, liver, spleen, lung, and kidney from each mouse were removed, photographed, and collected for later use.

### 2.11. Immunohistochemistry and H&E Staining

Tumor tissues were fixed using neutral formalin fixative for 24 h. Next, the tissues were dehydrated with graded ethanol, incubated with xylene (1330-20-7, Aladdin), and embedded into paraffin (P100936, Aladdin). The paraffin blocks were cut into 4 *μ*m sections, deparaffinized, rehydrated, and repaired using sodium citrate antigen retrieval solution (BB-2351-1, BestBio, Beijing, China). After the tissues were incubated with proliferating cell nuclear antigen (PCNA) antibody (1 : 1000, ab92552, Abcam, Cambridge, UK) and cleaved caspase 3 antibody (1 : 100, ab2302, Abcam) overnight at 4°C, they were washed with PBS and incubated with goat anti-rabbit IgG-HRP-conjugated secondary antibody (CW0103S, CWBIO, Taizhou, China) and diaminobenzidine (36201ES03, YEASEN, Shanghai, China). After sealing the tissues with neutral gum (BB-23474-1, BestBio), cell positive for PCNA and cleaved caspase 3 were observed under an inverted microscope. Tumor and five visceral tissues (heart, liver, spleen, lung, and kidney) were fixed, dehydrated, embedded, sectioned, and stained with hematoxylin-eosin solution (G1120, Solarbio), and the efficacy and safety of liensinine treatment were assessed.

### 2.12. Western Blotting

The protein in the SaOS-2, 143B, and hFOB 1.19 cells as well as in the tumor tissue was isolated by the NP-40 Lysis Buffer (N8032, Solarbio) containing PMSF (BB-3341-1, BestBio) and protease inhibitor mixture (P6730, Solarbio). Then, the protein concentration was quantified using a BCA kit (PC0020, Solarbio). 20 *μ*g of protein was electrophoresed inside the SDS-PAGE gel (P1200, Solarbio) and further transferred onto the PVDF membrane (YA1701, Solarbio) which further blocked with Western blot blocking buffer (BB-3512-1, BestBio) at room temperature for 2 h. Then, the membrane was, respectively, soaked overnight under the primary antibodies at 4°C: Bcl-2 (1 : 1000, ab32124, Abcam), Bax (1 : 2000, ab32503, Abcam), cleaved caspase 3 (1 : 2000, ab2302, Abcam), cleaved PARP1 (1 : 2000, ab32064, Abcam), Cyclin D1 (1 : 1000, ab16663, Abcam), p-STAT3 (1 : 3000, ab76315, Abcam), STAT3 (1 : 5000, ab119352, Abcam), p-JAK2 (1 : 4000, ab32101, Abcam), JAK2 (1 : 5000, ab108596, Abcam), and *β*-actin (1 : 1000, ab8226, Abcam). On the second day, the membrane was incubated with goat anti-rabbit IgG-HRP-conjugated secondary antibody (1 : 10000, ab205718, Abcam) or goat anti-mouse IgG-HRP-conjugated secondary antibody (1 : 10000, ab205719, Abcam) for 2 h at room temperature. The proteins were visualized by enhanced chemiluminescence (ECL) (BB-3501-2, BestBio) according to the manufacturer's protocol.

### 2.13. Routine Blood Tests and Liver/Renal Function Tests

After treatment, mice were anesthetized by intraperitoneal injection of 3% pentobarbital sodium, and eyeballs were removed for blood collection. 20 *μ*L whole blood was added into the EP tube containing 1 mL anticoagulant (1.5 mg/ml EDTA dipotassium salt dihydrate, Macklin, Shanghai, China) for routine blood test at the Animal Experimental Center, Peking University Health Science Center. At the same time, the blood in other EP tubes was placed at room temperature for 2 h, and then, the supernatant was taken and centrifuged at room temperature (3000 rpm, 10 min). About 200 *μ*L serum was collected for liver and renal function detection in the above institution.

### 2.14. Statistical Analysis

Statistical significances for independent data were assessed using an unpaired Student's *t*-test. For multiple comparisons, one-way ANOVA was used to analyze the data with post hoc Bonferroni correction. The data were analyzed using the IBM SPSS Statistics 22.0 software (IBM, Armonk, NY, USA), and the results were depicted as mean ± standard deviation (SD). Differences were considered significant at *P* < 0.05.

## 3. Results

### 3.1. Liensinine Inhibits the Proliferation of OS Cells but Not of the Human Normal Osteoblasts

**Figure 1:**
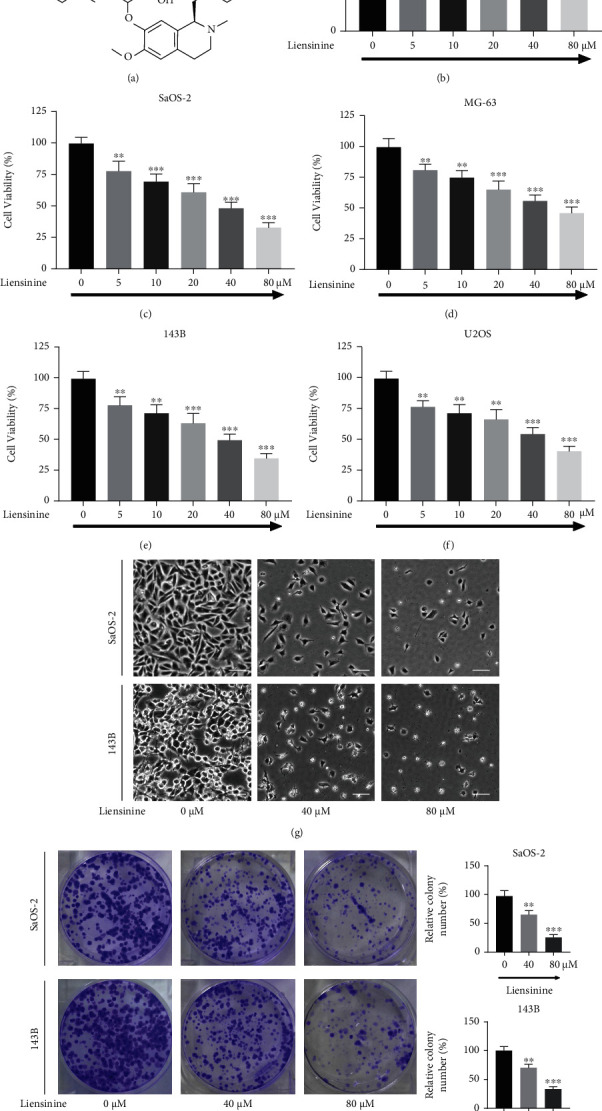
Liensinine inhibited the viability and proliferation of osteosarcoma cells. (a) The chemical structure of liensinine. (b) The viability of human normal osteoblasts (hFOB 1.19) after being treated with liensinine for 24 h was detected by CCK-8 assay. (c–f) The viability of human osteosarcoma cells (SaOS-2, MG-63, 143B, and U2OS) after being treated with liensinine for 24 h was detected by CCK-8 assay. (g) Changes in cell morphology of SaOS-2 and 143B cells were observed after liensinine treatment for 24 h; bar scale = 50 *μ*m. (h) The proliferation of SaOS-2 and 143B cells after being treated with liensinine was detected by colony formation assay. *n* = 3 in each group. The columns and errors bars represent means and SD. ^∗^*P* < 0.05, ^∗∗^*P* < 0.01, and ^∗∗∗^*P* < 0.001 vs. 0 *μ*M.

Liensinine (Figure 1(a)) was used to treat human normal osteoblasts (hFOB 1.19) and human OS cells (SaOS-2, MG-63, 143B, and U2OS). Cell viability was evaluated by the CCK-8 assay, which showed that liensinine at 5 *μ*M, 10 *μ*M, 20 *μ*M, 40 *μ*M, and 80 *μ*M did not inhibit the viability of hFOB 1.19 cells (Figure 1(b)); however, it significantly reduced the viability of SaOS-2 (Figure 1(c)), MG-63 (Figure 1(d)), 143B (Figure 1(e)), and U2OS (Figure 1(f)) cells. The viability of these cells reduced significantly after treatment with 5 *μ*M liensinine for 24 h (*P* < 0.01). The SaOS-2 and 143B cell lines showed the most significant effect of inhibition and were used for subsequent assays. After treated with 80 *μ*M liensinine for 24 h, their viability decreased to about a third of the control. Based on this result, we selected 40 *μ*M and 80 *μ*M liensinine to treat the cells in the following experiments. After exposure to 40 *μ*M and 80 *μ*M liensinine, morphological changes such as cell shrinkage, deformation, and cytoplasmic vesicles of SaOS-2 and 143B cells were observed by an optical microscope (Figure 1(g)). The results of the colony formation assay also indicated that liensinine inhibited cell proliferation and cloning efficiencies of SaOS-2 and 143B cells (Figure 1(h)). The number of colonies formed in both liensinine-treated groups was lower than that in the control group, and the inhibitory effect was clearly concentration-dependent. The cloning efficiencies of SaOS-2 at 40 *μ*M and 80 *μ*M compared with the control were 58.9% and 23.7% vs. 100.0% (40 *μ*M or 80 *μ*M vs. control: *P* = 0.004 or *P* < 0.0001), respectively. Correspondingly, the cloning efficiencies of 143B at 40 *μ*M and 80 *μ*M compared with the control were 67.4% and 30.3% vs. 100.0% (40 *μ*M or 80 *μ*M vs. control: *P* = 0.005 or *P* < 0.001), respectively.

### 3.2. Liensinine Induces Apoptosis of OS Cells

**Figure 2:**
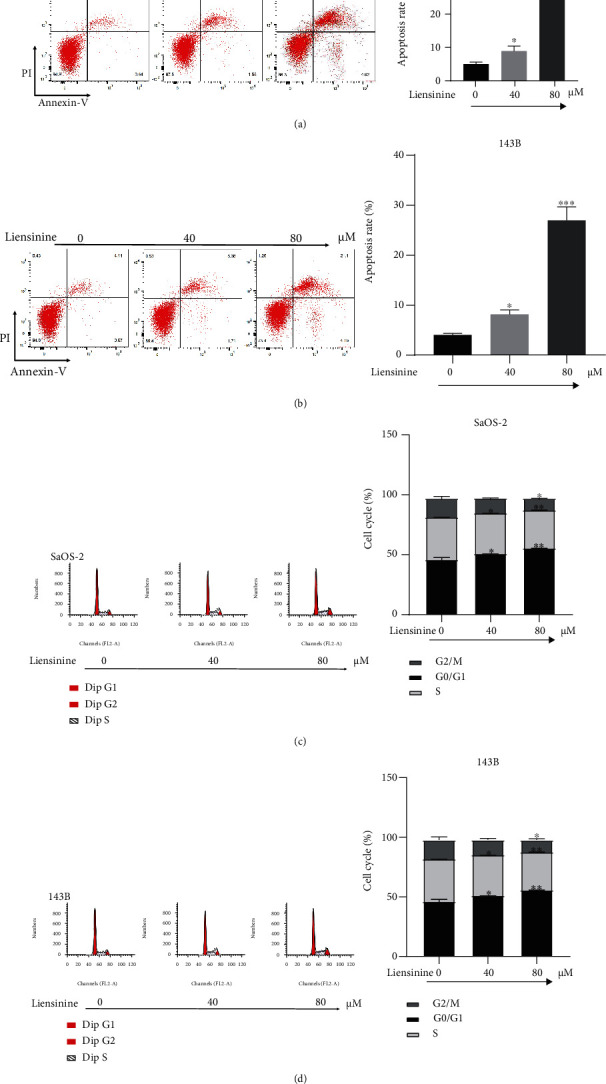
Liensinine induced the cell cycle arrest and apoptosis of osteosarcoma cells. (a, b) The apoptosis rates of SaOS-2 and 143B cells after being treated with liensinine were detected by FCM. (c, d) The cell cycle distribution of SaOS-2 and 143B cells after being treated with liensinine was detected by FCM. *n* = 3 in each group. ^∗^*P* < 0.05, ^∗∗^*P* < 0.01, ^∗∗∗^*P* < 0.001 vs. 0 *μ*M.

After treatment with 40 *μ*M and 80 *μ*M of liensinine, FCM showed that the total number of apoptotic cells in both the SaOS-2 and 143B cell lines increased significantly (Figures 2(a) and 2(b)). The apoptosis rates of SaOS-2 cells under 0, 40, and 80 *μ*M liensinine treatment were 4.8%, 9.6%, and 32.2%, respectively (40 or 80 vs. 0 *μ*M: *P* = 0.034 or *P* < 0.001). Similarly, the apoptosis rates of 143B cells were 4.3%, 8.7%, and 27.4%, respectively (40 or 80 vs. 0 *μ*M: *P* = 0.028 or *P* < 0.001).

### 3.3. Liensinine Induces Cycle Arrest in OS Cells

In both OS cell lines, liensinine increased the proportion of cells in G0/G1 phase and reduced the proportion of cells in S phase in a dose-dependent manner. The G0/G1 phase proportion of SaOS-2 cells under 0, 40, and 80 *μ*M liensinine treatment was 46.2%, 51.4%, and 55.9%, respectively (40 or 80 vs. 0 *μ*M: *P* = 0.031 or *P* = 0.004). Similarly, the G0/G1 phase proportion of 143B cells was 45.8%, 49.5%, and 54.1%, respectively (40 or 80 vs. 0 *μ*M: *P* = 0.012 or *P* = 0.001). Furthermore, liensinine at 80 *μ*M concentration significantly reduced the proportion of G2/M cells in SaOS-2 cells as compared to the corresponding control group (11.0% vs. 16.9%, *P* = 0.018) (Figures 2(c) and 2(d)). These results indicated that liensinine caused G0/G1 phase arrest, thereby inhibiting OS proliferation.

### 3.4. Liensinine Induces ROS Production and Causes Imbalance of GSSG/GSH

**Figure 3:**
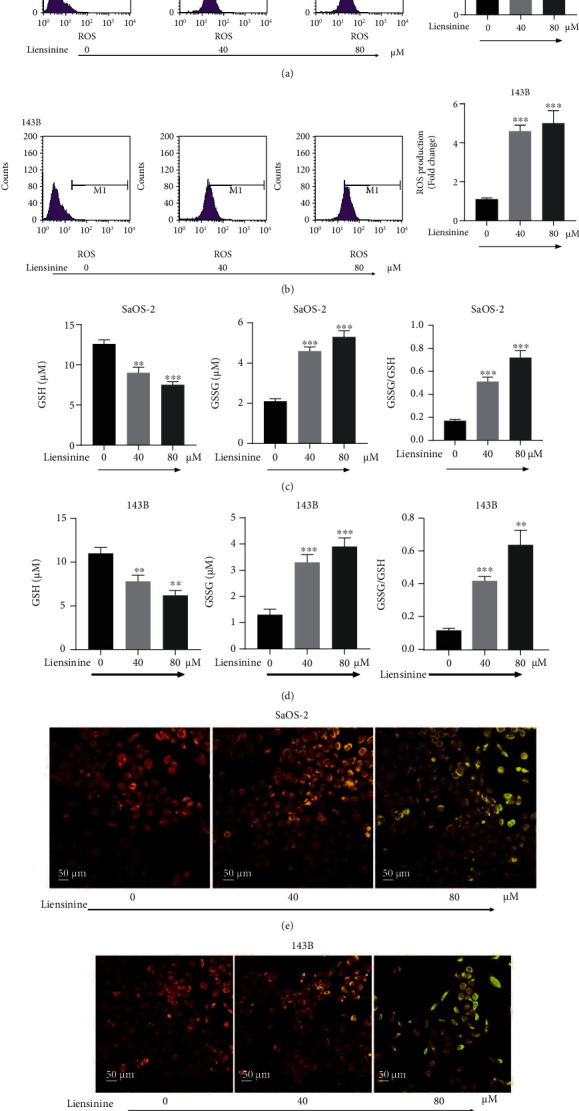
Liensinine-induced ROS production, GSSG/GSH imbalance, and MMP loss in osteosarcoma cells. (a, b) The ROS production in SaOS-2 and 143B cells after being treated with liensinine was detected by FCM. (c, d) The concentrations of GSH and GSSG, as well as the GSSG/GSH rate in SaOS-2 and 143B cells after being treated with liensinine ,were detected by colorimetry. (e, f) The MMP of SaOS-2 and 143B cells after being treated with liensinine was detected by JC-1 staining. *n* = 3 in each group. ^∗∗^*P* < 0.01 and ^∗∗∗^*P* < 0.001 vs. 0 *μ*M.

To evaluate if oxidative stress underlies the inhibitory effect of liensinine on OS cells, the ROS productions in SaOS-2 and 143B cells after liensinine treatment were examined by FCM analysis. Liensinine treatment substantially upregulated ROS production in both cell lines as compared to the control group (Figures 3(a) and 3(b)). The fold change of ROS production of SaOS-2 cells under 0, 40, and 80 *μ*M liensinine treatment was 1.0, 4.6 and 5.1, respectively (40 or 80 vs. 0 *μ*M: *P* < 0.001 or *P* < 0.001). Similarly, the fold change of ROS production of 143B cells was 1.0, 3.9, and 4.5, respectively (40 or 80 vs. 0 *μ*M: *P* < 0.001 or *P* < 0.001). In comparison, we evaluated the effect of the same concentration of liensinine on the intracellular ROS levels of hFOB 1.19 cells. The results showed that liensinine did not significantly affect the intracellular ROS levels of human normal osteoblasts (Figures S1(a)). In addition, we evaluated the changes in cellular GSH and GSSG levels in OS cell lines by colorimetric analysis. As compared to the control group, liensinine treatment significantly attenuated the GSH concentration (0, 40, and 80 *μ*M liensinine treatment of SaOS-2 cells: 12.6, 9.0, and 7.5 *μ*M, respectively, and 0, 40, and 80 *μ*M liensinine treatment of 143B cells: 11.1, 7.8, and 6.2 *μ*M, respectively); however, the GSSG concentration in both cell lines was elevated (0, 40, and 80 *μ*M liensinine treatment of SaOS-2 cells: 2.1, 4.6, and 5.3 *μ*M, respectively, and 0, 40, and 80 *μ*M liensinine treatment of 143B cells: 1.3, 3.3, and 3.9 *μ*M, respectively), thereby leading to a significantly increased GSSG/GSH ratio (0, 40, and 80 *μ*M liensinine treatment of SaOS-2 cells: 0.17, 0.51, and 0.71, respectively, and 0, 40, and 80 *μ*M liensinine treatment of 143B cells: 0.12, 0.42, and 0.64, respectively) (Figures 3(c) and 3(d)). These results indicated that liensinine could enhance ROS production and cause an imbalance of GSH/GSSG antioxidant system in OS cells.

### 3.5. Liensinine Induces MMP Loss

MMP was determined using JC-1 staining and was indicative of the extent of mitochondrial oxidative damage in OS cells. As shown in Figures 3(e) and 3(f), after exposure to 40 *μ*M and 80 *μ*M liensinine, the merged fluorescence color of SaOS-2 and 143B cells transformed from red to green in a dose-dependent manner under fluorescence microscope. It was proved that JC-1 existed in the cytoplasm as monomer, indicating that liensinine could induce substantial MMP decrease or loss in OS cells.

### 3.6. Liensinine Regulates the Expressions of Apoptosis and Cycle Arrest-Related Factors and Activates the JAK2/STAT3 Pathway

**Figure 4:**
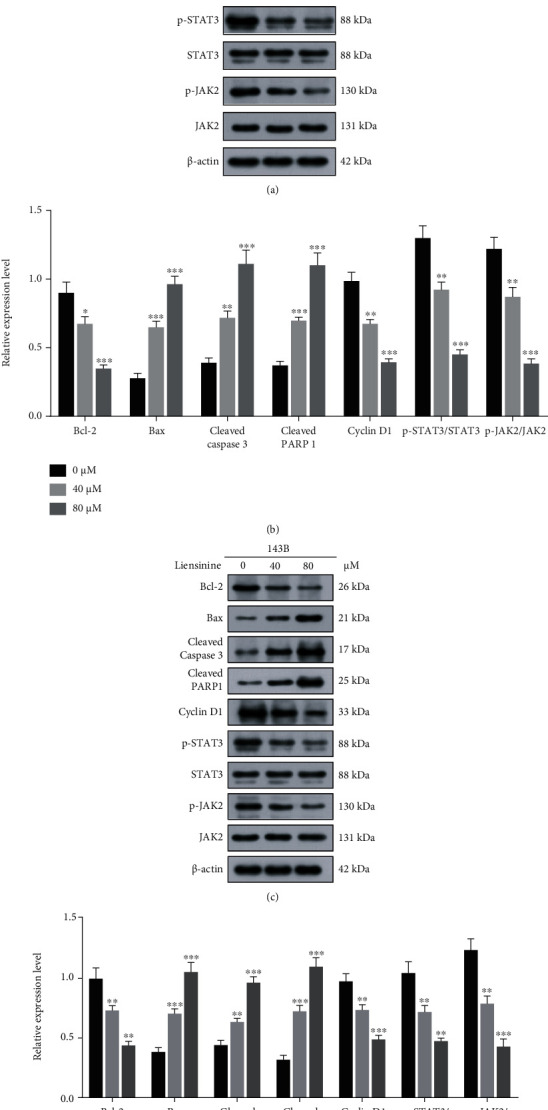
Liensinine regulated the expressions of factors related to apoptosis, cycle arrest, and JAK2/STAT3 pathway activation in osteosarcoma cells. (a, b) The expressions of factors related to apoptosis (Bcl-2, Bax, cleaved caspase 3, and cleaved PARP1), cycle arrest (Cyclin D1), and JAK2/STAT3 pathway activation (p-STAT3, STAT3, p-JAK2, and JAK2) in SaOS-2 cells after being treated with liensinine were detected by Western blotting. (c, d) The expressions of factors related to apoptosis, cycle arrest, and JAK2/STAT3 pathway activation in 143B cells after being treated with liensinine were detected by Western blotting. *n* = 3 in each group. ^∗^*P* < 0.05, ^∗∗^*P* < 0.01, and ^∗∗∗^*P* < 0.001 vs. 0 *μ*M.

To study the potential mechanism of liensinine affecting apoptosis and cell cycle progression, the expressions of factors related to apoptosis (Bcl-2, Bax, cleaved caspase 3, and cleaved PARP1) and cell cycle regulation (Cyclin D1) in OS cells were analyzed by Western blotting. Treatment with 40 and 80 *μ*M liensinine significantly upregulated the expressions of Bax, cleaved caspase 3, and cleaved PARP1, while downregulated those of Bcl-2 and Cyclin D1 (Figures 4(a)–4(d)), which were consistent with above findings for enhancing apoptosis and G0/G1 phase arrest. Many natural compounds affect tumor progression by suppressing activation of pathways involved in cell growth and proliferation, such as the JAK2/STAT3 pathway [20, 21]. We assessed the effect of liensinine on the expressions of proteins involved in the JAK2/STAT3 pathway by Western blotting. As shown in Figures 4(a)–4(d), treatment with 40 and 80 *μ*M liensinine significantly inhibited the expressions of p-STAT3 and p-JAK2, thereby reducing the ratio of p-STAT3/STAT3 and p-JAK2/JAK2. These results were negatively correlated with Bax expression and positively correlated with that of Bcl-2. Therefore, we reasonably speculated that liensinine-induced oxidative stress suppressed the activation of the JAK2/STAT3 pathway and further inhibited biological activity of OS cells. As the control, we also evaluated the effect of the same concentration of liensinine on apoptosis (cleaved caspase 3 and cleaved PARP1) and JAK2/STAT3 pathway activation (p-STAT3, STAT3, p-JAK2, and JAK2) of hFOB 1.19 cells. The results showed that the expressions of the above proteins remained unchanged upon liensinine treatment, which implied that, liensinine did not induce excessive apoptosis and activation of the JAK2/STAT3 pathway in human normal osteoblasts (Figures S1(b) and S1(c)).

### 3.7. ROS Scavenger Reverses the Effects of Liensinine on ROS Production, GSSG/GSH Imbalance, MMP Loss, Cell Apoptosis, and JAK2/STAT3 Pathway Activation

**Figure 5:**
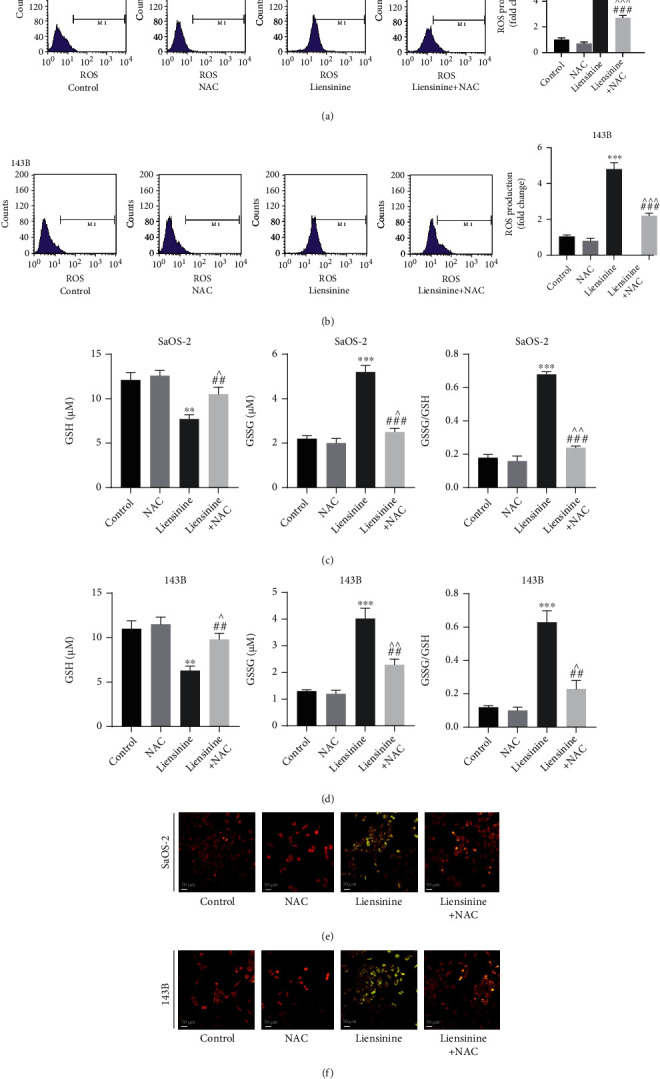
NAC reversed the effects of liensinine on ROS production, GSSG/GSH imbalance, and MMP loss in osteosarcoma cells. (a, b) The ROS production in SaOS-2 and 143B cells after being treated with NAC and liensinine was detected by FCM. (c, d) The concentrations of GSH and GSSG, as well as the GSSG/GSH rate in SaOS-2 and 143B cells after being treated with NAC and liensinine, were detected by colorimetry. (e, f) The MMP of SaOS-2 and 143B cells after being treated with NAC and liensinine was detected by JC-1 staining. *n* = 3 in each group. ^∗∗^*P* < 0.01 and ^∗∗∗^*P* < 0.001 vs. control; ^^^*P* < 0.05, ^^^^*P* < 0.01, and ^^^^^*P* < 0.001 vs. NAC; ^##^*P* < 0.01 and ^###^*P* < 0.001 vs. liensinine.

**Figure 6:**
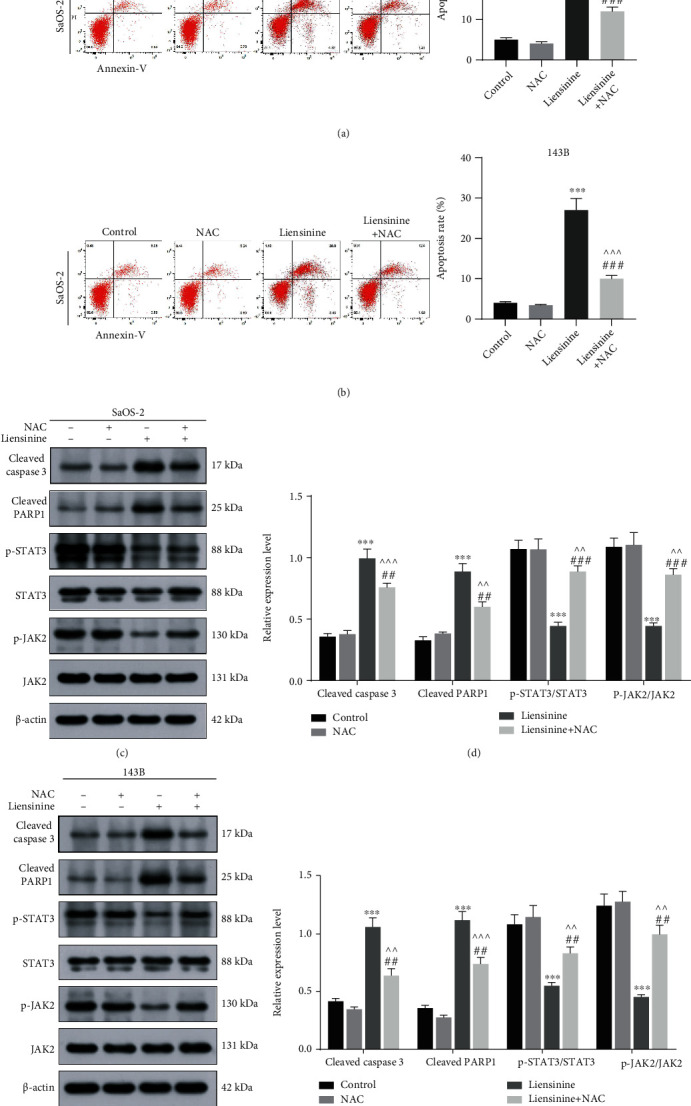
NAC reversed the effect of liensinine on apoptosis and the expressions of factors related to JAK2/STAT3 pathway activation in osteosarcoma cells. (a, b) The apoptosis rates of SaOS-2 and 143B cells after being treated with NAC and liensinine were detected by FCM. (c, d) The expressions of factors related to apoptosis (cleaved caspase 3 and cleaved PARP1) and JAK2/STAT3 pathway activation (p-STAT3, STAT3, p-JAK2, and JAK2) in SaOS-2 cells after being treated with liensinine were detected by Western blotting. (e, f) The expressions of factors related to apoptosis and JAK2/STAT3 pathway activation in 143B cells after being treated with liensinine were detected by Western blotting. *n* = 3 in each group. ^∗∗∗^*P* < 0.001 vs. control; ^^^^*P* < 0.01 and ^^^^^*P* < 0.001 vs. NAC; ^##^*P* < 0.01 and ^###^*P* < 0.001 vs. liensinine.

To further validate the inhibiting effect of liensinine treatment mediated by oxidative stress on OS, the two cell lines were treated with a combination of an antioxidant (NAC) and liensinine. NAC reversed intracellular ROS productions in SaOS-2 and 143B cells induced by 40 and 80 *μ*M liensinine (liensinine vs. liensinine+NAC treatment of SaOS-2 cells: 5.5 vs. 2.7, *P* < 0.001 and liensinine vs. liensinine+NAC treatment of 143B cells: 4.8 vs. 2.2, *P* < 0.001) (Figures 5(a) and 5(b)); NAC also reversed the decrease of GSH (liensinine vs. liensinine+NAC treatment of SaOS-2 cells: 7.7 vs. 10.5 *μ*M, *P* = 0.007 and liensinine vs. liensinine+NAC treatment of 143B cells: 6.3 vs. 9.8, *P* = 0.002) and the increase of GSSG (liensinine vs. liensinine+NAC treatment of SaOS-2 cells: 5.2 vs. 2.5, *P* < 0.001 and liensinine vs. liensinine+NAC treatment of 143B cells: 4.0 vs. 2.3, *P* = 0.003) in both cell lines (Figures 5(c) and 5(d)). NAC remarkably alleviated the loss of MMP induced by liensinine treatment, corresponding to the change of merged fluorescence color from green to red (Figures 5(e) and 5(f)). In addition, FCM showed that NAC significantly inhibited the apoptosis of OS cells induced by liensinine treatment (Figures 6(a) and 6(b)). The apoptosis rates of SaOS-2 cells under liensinine and liensinine+NAC treatment were 33.8% and 12.4% (liensinine vs. liensinine+NAC: *P* < 0.001); the apoptosis rates of 143B cells under liensinine and liensinine+NAC treatment were 27.3% and 10.1% (liensinine vs. liensinine+NAC: *P* < 0.001). On protein expression, NAC reversed the liensinine-induced upregulation of the expressions of cleaved caspase 3 and cleaved PARP1 (Figures 6(c)–6(f)), consistent with the decline in the apoptosis rates in OS cells; NAC also reversed the liensinine-induced downregulation of p-STAT3 and p-JAK2 and significantly increased the ratio of p-STAT3/STAT3 and p-JAK2/JAK2 (Figures 6(c)–6(f)). Taken together, these results indicated that the antitumor effect and the activation of JAK2/STAT3 pathway upon liensinine treatment were mediated by excessive oxidative stress.

### 3.8. Liensinine Inhibits OS Growth *In Vivo*

**Figure 7:**
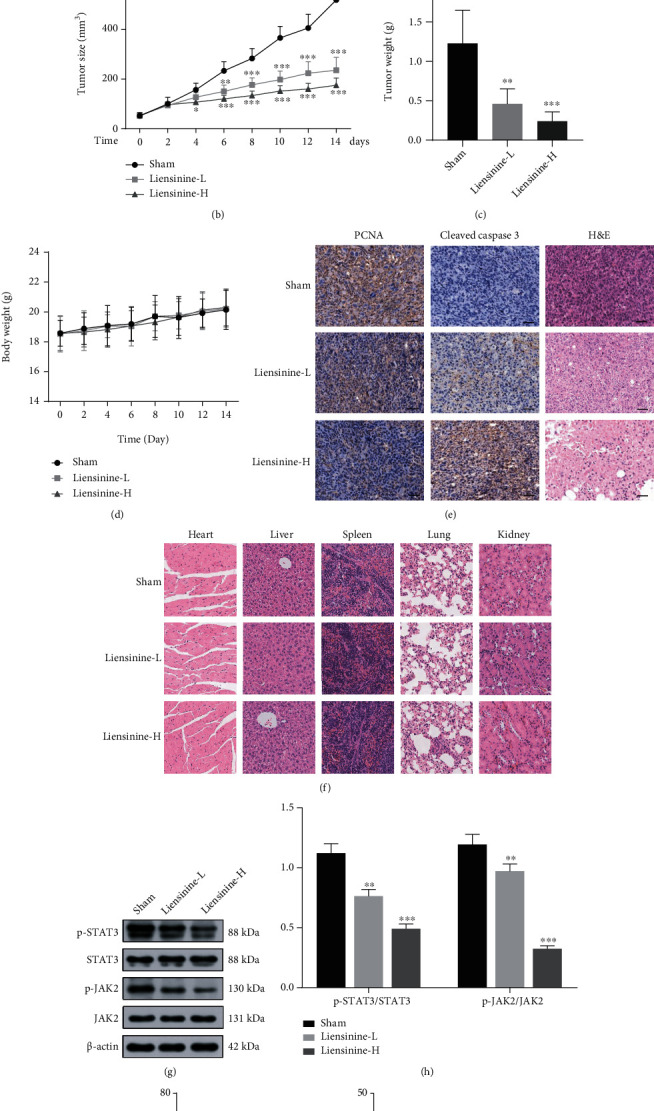
Liensinine inhibited growth and induced apoptosis of subcutaneous xenografts *in vivo*. (a) The final subcutaneous xenografts were photographed. (b) The tumor volumes were recorded for 14 days. (c) The final subcutaneous tumors were weighed. (d) The body weights of mice were weighed. (e) The expressions of PCNA and cleaved caspase 3 in tumor tissues were analyzed by immunohistochemical staining, and the tumor structure destruction was demonstrated by H&E staining; bar scale = 100 *μ*m. (f) The effect of liensinine on general organ structures was analyzed by H&E staining of heart, liver, spleen, lung, and kidney; bar scale = 100 *μ*m. (g) The expressions of p-STAT3, STAT3, p-JAK, and JAK in tumor tissues were detected by Western blotting. (h) The ratios of p-STAT3/STAT3 and p-JAK/JAK were calculated based on the strip gray values of Western blotting. (i, j) Liver and renal function of mice post 2-week indicated treatment were tested. Representative items: ALT: alanine aminotransferase; Cr: creatinine. *n* = 6 in each group of (b–d, i, j). *n* = 3 in each group of (g). ^∗^*P* < 0.05, ^∗∗^*P* < 0.01, and ^∗∗∗^*P* < 0.001 vs. sham.

To evaluate the effect of liensinine on OS growth *in vivo*, we established subcutaneous-xenograft models using 143B cells. The mice were treated with liensinine at doses of 15 mg/kg and 30 mg/kg for two weeks. The photos of excised tumors from each group are shown in Figure 7(a). Changes in tumor volume during the two-week treatment are shown in Figure 7(b); the final tumor weights are shown in Figure 7(c). Liensinine treatment could significantly slow down the growth of tumor volume and weight in a dose-dependent manner. The body weight changes of mice during two weeks were similar among the groups, as shown in Figure 7(d), suggesting that liensinine exerted no systemic toxicity in mice. PCNA immunohistochemical staining was used to evaluate the proliferation status of tumor cells. After liensinine treatment, the proportion of PCNA positive cells decreased substantially, while the number of cleaved caspase 3 positive cells increased in tumor tissues. H&E staining results clearly demonstrated serious tumor cell damage by liensinine, characterized by the reduction of cells with hyperchromatic nuclei and the proliferation of fibrous tissues (Figure 7(e)). In addition, H&E staining results of the heart, liver, spleen, lung, and kidney excised from the mice showed that liensinine had no negative effect on internal organs as compared to the control group (Figure 7(f)). Through Western blot analysis of tumor tissues, we found that liensinine treatment significantly downregulated the expressions of p-STAT3 and p-JAK2, thereby causing a significant reduction in the ratio of p-STAT3/STAT3 and p-JAK2/JAK2 (Figures 7(f) and 7(g)). These results were consistent with the suppression of JAK2/STAT3 pathway by liensinine *in vitro*. In addition, no significant change was found in the routine blood test as well as the liver and renal function blood test (Figures 7(i) and 7(j); Figure S1(d)–S1(g)). Thus, the administration of liensinine was biocompatible in mice.

## 4. Discussion

Traditional Chinese medicine (TCM) has been widely used to treat various diseases for thousands of years. Among them, liensinine, a natural isoquinoline alkaloid, can be isolated from a Chinese medicinal herb (Nelumbo nucifera Gaertn) and possesses several medicinal benefits [6–8]. Increasing evidence shows that liensinine may be a potential antitumor agent, and its efficacy against human colorectal, gallbladder, and breast cancers has been validated by previous studies [6, 9, 10]. As a malignant tumor originating from mesenchymal tissue, OS has faster progression, earlier hematogenous metastasis, and fewer targeted drugs available than the aforementioned cancer types. Thus, it requires more radical resection as it causes more misery to the patients [22]. Therefore, it is important to develop efficient and safe pharmacotherapy for OS. To date, the effect of liensinine in OS has not been reported, both *in vitro* and *in vivo*.

In this study, liensinine was found to significantly reduce the viabilities and cloning efficiencies of multiple OS cell lines in a dose-dependent manner; it exerted no negative effect on human normal osteoblasts, even at a relatively higher dose. Tumor cells usually have higher metabolic activities and ROS levels as compared to the normal cells and are more vulnerable to liensinine-induced ROS insults [17, 23]. The mechanism underlying liensinine-mediated elevation in oxidative stress in tumor cells requires further study. In addition, tumor cells show higher cellular uptake of natural compounds but have lower intracellular glutathione levels than normal cells, which may be related to the selective cytotoxic effect of liensinine on OS cells [24, 25]. The results of flow cytometry analysis indicated that liensinine could substantially block G0/G1 progression into S phase and promote apoptosis in OS cells, consistent with the previous studies that reported the apoptosis-induction effect of liensinine on other cancer cell types [9, 10, 14]. We also confirmed the antitumor activity of liensinine in xenografted-tumor mice, which further validated our *in vitro* findings as indicated by the subcutaneous tumor sizes and immunohistochemical results post 2-week treatment. Moreover, according to the H&E staining of the visceral slices, routine blood test, and liver and renal function test, we concluded that the appropriate dosage of liensinine was safe and reliable for *in vivo* application.

To validate the above findings at the molecular level, we performed Western blot analysis. The imbalance in the expressions of Bax and Bcl-2 is involved in mitochondrial apoptosis, inducing the expression of cleaved caspase 3 and promoting tumor apoptosis [26]. Once caspase 3 is cleaved and activated, cascading effects ensue, and the apoptosis process is irreversible. Therefore, caspase 3 is considered to be a key factor in triggering apoptosis [27]. PARP is a nuclear protein that is activated by DNA damage and participates in DNA repair. However, it is also a substrate of the caspases, which can lose enzymatic activity and accelerate apoptosis after cleavage [28, 29]. Cyclin D1 is a regulatory subunit of cyclin-dependent kinases (CDKs) that promotes G0/G1 progression [30]. Hence, changes in expressions of Bcl-2, Bax, cleaved caspase 3, cleaved PARP, and Cyclin D1 reflect the apoptosis and cell cycle status. After liensinine treatment, Bax, cleaved caspase 3, and cleaved PARP were found to be upregulated, while those of Bcl-2 and Cyclin D1 were downregulated in OS cells, which indicated that liensinine may be a potential agent for inhibiting OS progression.

The key roles of ROS in biological processes include cell signaling, biosynthetic processes, and host defense [31]. A mild elevation of ROS levels and ROS scavenging rate within tumor cells, termed as “mild oxidative” stress, is their normal physiological state and is associated with the activation of oncogenic pathways [32, 33]. However, sustained accumulation of ROS, especially free radicals, will cause damage to DNA, lipids, and proteins, eventually leading to adverse cellular outcomes [11–13]. Many drugs exert potent cytotoxic effects through this mechanism, for example, Glaucocalyxin A inhibits OS progression by inducing excessive oxidative stress [11]. GSH is a potent antioxidant that eliminates intracellular ROS through a redox reaction, by itself being oxidized to GSSG [34]. The imbalance of GSSG/GSH system reflects a notable deficiency in ROS scavenging capacity [11, 35]. To explore whether liensinine can promote ROS production, the levels of ROS, GSSG, and GSH in OS cells were evaluated. The ROS and GSSG levels were found to be upregulated while that of GSH was downregulated after treatment, suggesting that liensinine could induce excessive oxidative stress in OS cells. With the decrease of MMP, ROS increase and are released from mitochondria into the cytoplasm, further resulting in excessive oxidative stress, which subsequently triggers more release of ROS from the neighboring mitochondria, ultimately leading to cell death [36]. A decline in MMP is therefore a hallmark event in the early stage of apoptosis [37]. We found significant MMP loss in liensinine-treated tumor cells by JC-1 staining, confirming the occurrence of oxidative stress damage. Moreover, pretreatment with NAC, an antioxidant or ROS scavenger, could significantly reduce ROS production and apoptosis of OS cells stimulated by liensinine, which indicated that the liensinine-induced oxidative stress injury was directly responsible for the inhibition of OS survival.

**Figure 8:**
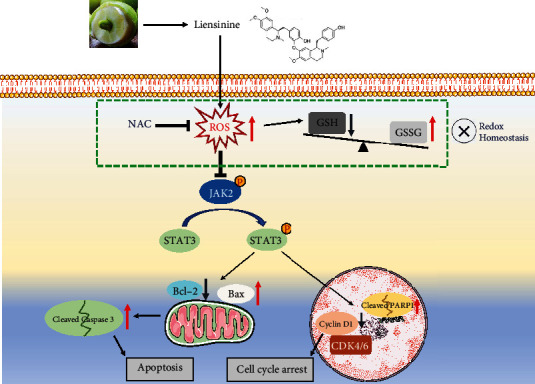
Proposed model for the role of liensinine-induced ROS accumulation in the osteosarcoma treatment. Increased ROS generation can lead to oxidant and antioxidant imbalance and suppress the activation of JAK2/STAT3 pathway, promoting OS cell cycle arrest and apoptosis.

Previous studies have demonstrated that the biological activities of tumor cells are regulated by multiple signaling pathways including the JAK2/STAT3 cascade [20, 21, 38, 39]. Activation of the JAK2/STAT3 pathway can promote cell survival and proliferation, inhibit apoptosis, and is implicated in the occurrence and development of various cancers [40, 41]. Its antiapoptotic effect may be realized by reducing the Bax/Bcl-2 apoptotic switch ratio and inhibiting the activation of key proapoptotic enzymes in cells [42, 43]. In this study, we first reported that liensinine could suppress the activation of the JAK2/STAT3 pathway in tumor cells. Furthermore, excessive intracellular ROS can induce cell cycle arrest and apoptosis [32, 44]. Therefore, we reasonably speculated that the liensinine-induced intracellular ROS accumulation may suppress the activation of the JAK2/STAT3 pathway, thereby inhibiting the proliferation and promoting the apoptosis of OS cells. The mediating role of ROS was confirmed when NAC was found to significantly reverse the liensinine-induced JAK2/STAT3 pathway blockade. Together, the results suggested that liensinine could exert antitumor effect by affecting JAK2/STAT3 pathway activation through excessive oxidative stress (Figure 8). The finding that the JAK2/STAT3 pathway could be blocked by excessive ROS was consistent with the tumor-related reports [11, 13, 45], but contrary to the conclusions of some inflammation-related studies [46, 47]. This may be due to differential roles and mechanisms of ROS in tumor cells and macrophages, which requires further exploration.

In conclusion, the present study showed that the liensinine-induced ROS production could suppress the activation of the JAK2/STAT3 pathway and inhibited the OS growth both *in vivo* and *in vitro*. Our findings provided a new rationale for subsequent academic and clinical research on OS treatment.

## Data Availability

The data collected for the study are available from the corresponding author upon request.

## Abbreviations

OS: Osteosarcoma
CCK-8: Cell Counting Kit-8
ROS: Reactive oxygen species
GSH: Glutathione
GSSG: Glutathione disulfide
MMP: Mitochondrial membrane potential
NAC: N-Acetyl cysteine
FCM: Flow cytometry
SPF: Specific pathogen free
PCNA: Proliferating cell nuclear antigen
p-JAK2: Phosphorylated JAK2
p-STAT3: Phosphorylated STAT3
SD: Standard deviation
TCM: Traditional Chinese medicine
CDKs: Cyclin-dependent kinases
WBC: White blood cells
RBC: Red blood cells
ALT: Alanine aminotransferase
AST: Aspartate aminotransferase
Cr: Creatinine
BUN: Blood urea nitrogen.

## Supplementary Materials

Supplementary Materials Figure S1: effect of liensinine on ROS production and JAK2/STAT3 pathway activation in hFOB 1.19 cells and hematological evaluation of its biocompatibility.
