# Supplementary material for: Liensinine Inhibits Osteosarcoma Growth by ROS-Mediated Suppression of the JAK2/STAT3 Signaling Pathway
Source: Oxid Med Cell Longev. 2022 Jan 25;2022:8245614. doi: 10.1155/2022/8245614 (PMC8807040; doi:10.1155/2022/8245614)
Supplement: Supplementary Materials — Figure S1: effect of liensinine on ROS production and JAK2/STAT3 pathway activation in hFOB 1.19 cells and hematological evaluation of its biocompatibility. [file 8245614.f1.docx]

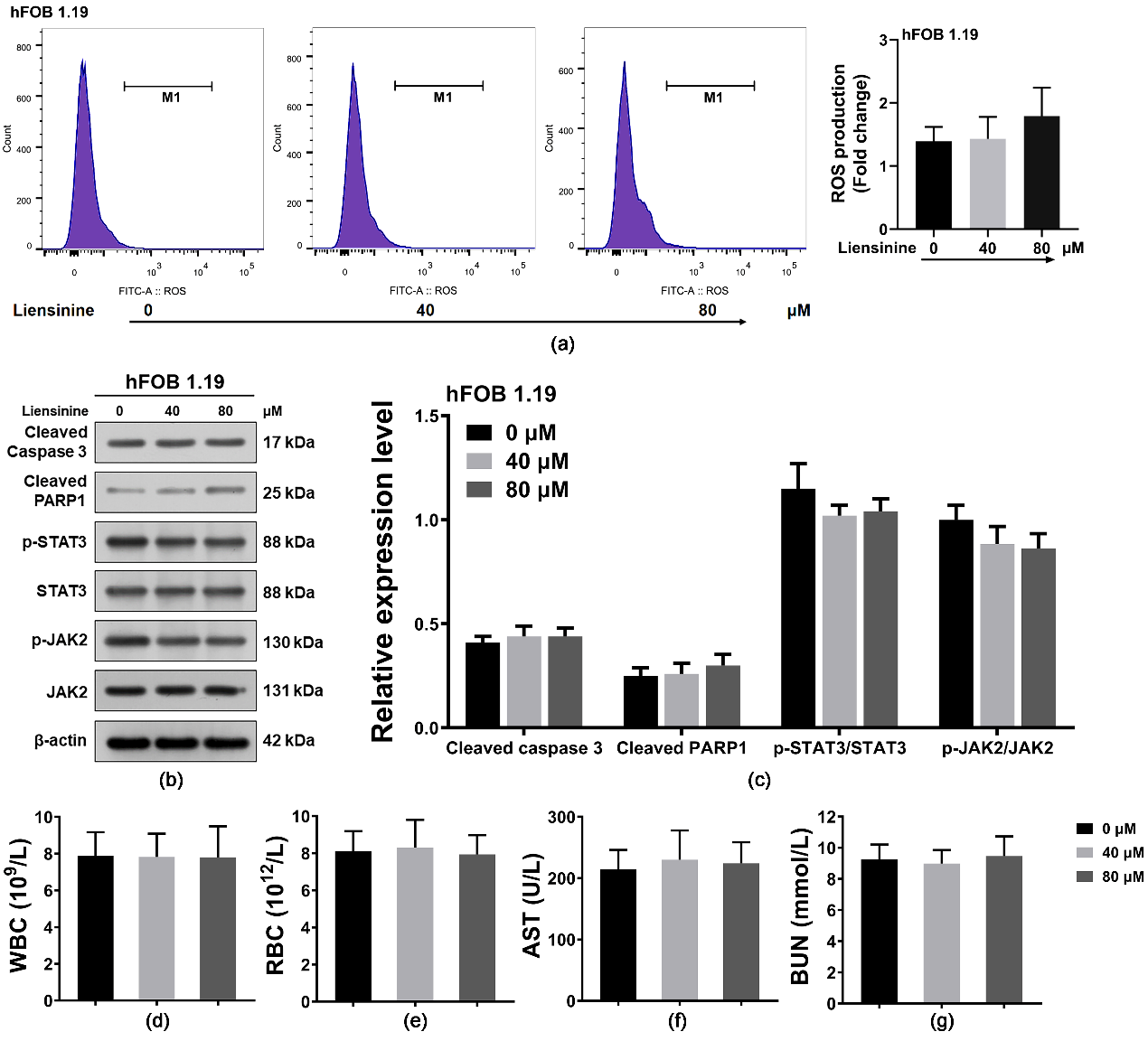


**Figure S1 Effect of Liensinine on ROS production and JAK2/STAT3 pathway in hFOB 1.19 cells, and hematological evaluation of its systemic toxicity *in vivo*.**

(a) The ROS production in hFOB 1.19 cells after being treated with liensinine was detected by FCM. (b-c) The expression of factors related to apoptosis (Cleaved caspase 3, Cleaved PARP1) and JAK2/STAT3 pathway activation (p-STAT3, STAT3, p-JAK2, JAK2) in hFOB 1.19 cells after being treated with liensinine was detected by western blotting. (d-e) Routine blood tests of mice post 2-week indicated treatment were performed. Representative items: WBC, white blood cells; RBC, red blood cells. (f-g) Liver and renal function of mice post 2-week indicated treatment were tested. Representative items: AST, aspartate aminotransferase; BUN, blood urea nitrogen. n = 3 in each group of Figure S1(a) and S1(c). n = 6 in each group of Figure S1(d)-S1(g).
